# Clinical and neurocognitive profiles of a combined clinical high risk for psychosis and clinical control sample: latent class analysis

**DOI:** 10.1192/bjo.2024.815

**Published:** 2024-12-05

**Authors:** Miriam Stüble, Frauke Schultze-Lutter, Michael Kaess, Maurizia Franscini, Nina Traber-Walker, Petra Walger, Benno G. Schimmelmann, Kai Vogeley, Joseph Kambeitz, Jochen Kindler, Chantal Michel

**Affiliations:** University Hospital of Child and Adolescent Psychiatry and Psychotherapy Bern, University of Bern, Switzerland; and Graduate School for Health Sciences, University of Bern, Switzerland; University Hospital of Child and Adolescent Psychiatry and Psychotherapy Bern, University of Bern, Switzerland; Department of Psychiatry and Psychotherapy, Medical Faculty, Heinrich-Heine University, Germany; and Department of Psychology, Faculty of Psychology, Airlangga University, Indonesia; University Hospital of Child and Adolescent Psychiatry and Psychotherapy Bern, University of Bern, Switzerland; and Department of Child and Adolescent Psychiatry, Centre for Psychosocial Medicine, University of Heidelberg, Germany; Department of Child and Adolescent Psychiatry and Psychotherapy, University of Zurich, Switzerland; Department of Psychiatry and Psychotherapy, Medical Faculty, Heinrich-Heine University, Germany; University Hospital of Child and Adolescent Psychiatry and Psychotherapy Bern, University of Bern, Switzerland; and University Hospital of Child and Adolescent Psychiatry, University Hospital Hamburg-Eppendorf, Germany; Department of Psychiatry and Psychotherapy, Faculty of Medicine and University Hospital Cologne, University of Cologne, Germany; and Cognitive Neuroscience (INM3), Institute of Neuroscience and Medicine, Research Center Jülich, Germany; Department of Psychiatry and Psychotherapy, Faculty of Medicine and University Hospital Cologne, University of Cologne, Germany; University Hospital of Child and Adolescent Psychiatry and Psychotherapy Bern, University of Bern, Switzerland

**Keywords:** Psychotic symptoms, basic symptoms, neurocognition, clinical heterogeneity, early intervention

## Abstract

**Background:**

The clinical high-risk (CHR) state for psychosis demonstrates considerable clinical heterogeneity, presenting challenges for clinicians and researchers alike. Basic symptoms, to date, have largely been ignored in explorations of clinical profiles.

**Aims:**

We examined clinical profiles by using a broader spectrum of CHR symptoms, including not only (attenuated) psychotic, but also basic symptoms.

**Method:**

Patients (*N* = 875) of specialised early intervention centres for psychosis in Germany and Switzerland were assessed with the Schizophrenia Proneness Instruments and the Structured Interview for Psychosis-Risk Syndromes. Latent class analysis was applied to CHR symptoms to identify clinical profiles. Additionally, demographics, other symptoms, current non-psychotic DSM-IV axis I disorders and neurocognitive variables were assessed to further describe and compare the profiles.

**Results:**

A three-class model was best fitting the data, whereby basic symptoms best differentiated between the profiles (η^2^ = 0.08–0.52). Class 1 had a low probability of CHR symptoms, the highest functioning and lowest other psychopathology, neurocognitive deficits and transition-to-psychosis rate. Class 2 had the highest probability of basic and (attenuated) positive symptoms (excluding hallucinations), lowest functioning, highest symptom load, most neurocognitive deficits and highest transition rate (55.1%). Class 3 was mostly characterised by attenuated hallucination, and was otherwise intermediate between the other two classes. Comorbidity rates were comparable across classes, with some class differences in diagnostic categories.

**Conclusions:**

Our profiles based on basic and (attenuated) psychotic symptoms provide clinically useful entities by parsing out heterogeneity in clinical presentation. In future, they could guide class-specific intervention.

The importance of early detection and intervention in psychosis, in terms of an indicated prevention in clinical high risk for psychosis (CHR) states, has been widely recognised and established internationally.^1^ For the purpose of defining a CHR state, two sets of CHR criteria are used: the ultra-high risk (UHR) and the basic symptoms criteria.^2,3^ For their spontaneous, immediate recognition by patients as disturbances of their own mental processes, basic symptoms are distinct from the symptoms that define the UHR criteria (i.e. attenuated psychotic symptoms (APS) or brief intermittent psychotic symptoms (BIPS)) and from more persistent and obvious psychotic symptoms, in which reality testing is disturbed, at least to some degree. However, (attenuated) psychotic symptoms are considered to arise from basic symptoms, when there is an unfavourable environment or the symptom load is too high.^4^ Although early studies reported high transition rates of 25−35% for UHR criteria and up to 60% for basic symptom criteria to manifest psychosis at 4 years and beyond,^3,5^ more recent research, mainly with follow-ups of only 2–3 years, has yielded lower transition rates.^6^ Yet, in the majority of CHR patients, psychological distress continues to be significant, and many patients who do not transition to psychosis experience persistent APS, cognitive basic symptoms and low psychosocial functioning.^7^ Moreover, longitudinal findings indicate that experiencing APS (regardless of transition) increases the chances of developing or maintaining other mental disorders.^8^ Thus, independent of psychosis transition, CHR symptoms represent clinically relevant phenomena in need of treatment.^9,10^

## Phenomenological heterogeneity as a challenge

Next to CHR symptoms, the CHR state is accompanied by a range of comorbid psychopathological symptoms, neurocognitive deficits and impairments in psychosocial functioning,^2,3^ and similar to the heterogeneity of medium-term outcomes of CHR states and other psychiatric disorders,^11^ the clinical presentation of the CHR state itself demonstrates considerable phenomenological heterogeneity that presents challenges for clinicians and researchers alike.^12^ Thus, the identification of clinically meaningful homogenous subgroups is critical for clinical applicability and the definition of specific intervention targets.^13^ One common statistical approach to parsing out heterogeneity in psychiatric samples is latent class analysis (LCA).^14,15^ LCA is used to identify qualitatively different subgroups with independent symptom constellations and different associations with other clinically relevant impairments within populations who share certain outward characteristics, such as CHR samples.^16,17^ Across CHR studies, three to five subclasses have been found, and different classes of symptoms were associated with significant differences in the risk for conversion.^14,16,17^ However, few LCA studies included neurocognitive measures and none of them included basic symptoms across clinical profiles. And this despite the fact that neurocognitive deficits in CHR patients have been found to be especially pronounced in the domains of processing speed (which may be a relevant domain for interventions to enhance neurocognition in CHR samples) and memory-related tasks.^18,19^ Therefore, neurocognitive deficits may be a useful feature in differentiating subgroups, and will be assessed in the current study to help characterise clinical profiles. Furthermore, despite basic symptoms being part of the recommended CHR criteria of the European Psychiatric Association (EPA) guidance project,^3^ and considered the earliest subtle and subjectively experienced symptoms of psychosis,^20^ to date, they have been largely ignored in efforts at exploring clinical profiles. Incorporating the measurement of basic symptoms might provide novel and useful ways to meaningfully identify and differentiate between clinical profiles, and thus, is a worthy inclusion.

## Aims of the study

The aims of the current study were to identify clinical profiles by using a broader spectrum of CHR symptoms, including both basic symptoms and (attenuated) psychotic symptoms, and to describe the profiles in a clinically comprehensive manner, characterising them in terms of psychiatric comorbidities, other symptom ratings (i.e. negative symptoms), functioning and neurocognitive measures, to present clinically useful profiles by parsing out heterogeneity in clinical presentation, enhancing both early detection and intervention in patient populations (aged 8–40 years) of early detection services.

## Method

### Participants and procedures

For the current study, data from 875 patients were used. This sample comprised three subsamples: (a) patients between 8 and 40 years of age from the Early Recognition and Intervention Centre for Mental Crisis (FETZ) Bern (*n* = 257, 29.4%);^21^ (b) patients between 16 and 40 years of age from the FETZ Cologne (*n* = 157, 17.9%)^22^ and (c) patients between 8 and 17 years of age who were participating in the naturalistic study ‘Bi-national Evaluation of At-Risk Symptoms in Children and Adolescents’ (BEARS-Kid), which was conducted at the Universities of Bern, Zurich and Cologne (*n* = 461, 52.7%).^23,24^ The BEARS-Kid sample composed of patients meeting CHR criteria and clinical controls (i.e. in-patients with a primary diagnosis of attention-deficit hyperactivity disorder, eating disorders, anxiety (including obsessive–compulsive) disorders or autism spectrum disorder, not clinically suspected of developing psychosis).^25^ Written consent for the use of the data was obtained from all participants, and from legal guardians of participants under 18 years of age. Exclusion criteria included a past clinical diagnosis of any psychotic disorder according to DSM-IV and ICD-10 criteria, and insufficient German, French or English language skills. In the FETZ Bern and Cologne, assessments formed part of the routine diagnostic assessment procedure at entry. Within the BEARS-Kid study, psychologists received 3 months training in conducting the interviews before the commencement of the study, as well as weekly supervision by two of the authors (C.M. and F.S.-L.), to ensure excellent data quality.^23^ Patients at FETZ Cologne were supervised by F.S.-L., and those at FETZ Bern were supervised by either C.M. or F.S.-L.

All procedures comply with ethical standards of the relevant national and institutional committees on human experimentation and with the Helsinki Declaration of 1975, as revised in 2008. The human research ethics committee of the Canton Bern approved the coded clinical data to be used in scientific analyses of the FETZ Bern (identifier: PB_2016-01991) and approved the main ethics application for the BEARS-Kid study for all three centres (Bern: ID_PB_2016-02192; Zurich: 2010-0415/3; Cologne: ID 11-071). The FETZ Cologne was approved by the ethics committee of Cologne (ID 19-1618_1).

### Measures

#### Assessment of CHR symptoms

For UHR symptoms/criteria, the Structured Interview for Psychosis-Risk Syndromes (SIPS),^26^ a semi-structured interview that rates along four major symptom dimensions, was used: five positive, six negative, four disorganisation and four general items, each syndromally scored 0–6 according to severity and according to impact on behaviour and/or conviction level. APS and BIPS were defined as a score of 3–5 and 6, respectively, on the five positive items.

Basic symptoms were assessed with the Schizophrenia Proneness Instruments (SPI-A/SPI-CY)^27,28^ and rated on a scale from 0 to 6, according to their frequency of occurrence. Altogether, 14 cognitive and perceptual basic symptoms are included in the two basic symptoms criteria, Cognitive Disturbances (COGDIS) and Cognitive-Perceptive Basic Symptoms (COPER).^29^

For the present analyses, CHR symptoms were defined by the presence of any APS/BIPS and/or basic symptoms, irrespective of the onset/worsening and/or frequency requirements of related CHR criteria. For the purpose of this study, basic symptoms and SIPS positive symptom scores were dummy-coded before analysis. A basic symptoms score of 0 (absent) remained a 0 (basic symptoms absent), whereas scores from 1 to 6 were assigned a 1 (basic symptoms present). A SIPS positive symptom score from 0 to 2 was assigned a 0 (APS/BIPS absent), and scores from 3 to 6 were assigned a 1 (APS/BIPS present). Symptoms were only rated if the phenomenon in question was not fully and better explained by another nonpsychotic disorder, a somatic cause or psychotropic drug use.^26^

#### Assessment of comorbidities, other symptom ratings and functioning

For the purpose of characterising distinct clinical profiles, data regarding axis 1 disorders according to the DSM-IV (measured with the Mini-International Neuropsychiatric Interview (MINI/MINI-Kid), where available),^30^ negative, disorganised and general symptoms of the SIPS^26^ as well as current and highest past-year psychosocial functioning (measured with the Social and Occupational Functioning Assessment Scale (SOFAS))^31^ were used. The SOFAS is a rating scale based on the DSM-IV fourth axis and the clinician's judgement of overall level of functioning. Ranging from 0 to 100, with lower scores indicating lower functioning, it is a global rating of current functioning independent of the overall severity of the individual's psychological symptoms.^32^ Psychometric properties including the interrater reliability and construct validity are good.^33,34^ Scores between 31 and 70 refers to manifest disabilities of different degrees and scores below 30 reflect poor functioning with the need for intensive support or supervision.^35^

#### Assessment of neurocognition

The test battery for each neurocognitive domain (verbal memory, processing speed, (verbal) executive functions and spatial memory) assessed can be found in Supplementary Table 1 available at https://doi.org/10.1192/bjo.2024.815. Neurocognitive deficits were defined based on previous studies,^36^ relative to the normative data provided for each test. More than 1 s.d. below mean, a *T*-score below 40 or a percentile below 16 were rated as 1 (deficient). Scores higher than these norms were coded as 0 (not deficient). According to the DSM-5 severity dimension, a neurocognitive deficit using these criteria corresponds to at least a ‘moderate cognitive deficit’, i.e. ‘a clear reduction in cognitive function below expected for age and socioeconomic status’.^31^

### Statistical analyses

R (version 4.2 for Windows; see https://posit.co/downloads/) and RStudio (version 2022.07.0 for Windows; see https://cran.r-project.org/bin/windows/base/) were used to conduct the analyses. Number of classes was not estimated *a priori*. To identify the best-fitting LCA model, a one-class model was initially fitted, and subsequent classes were added in accordance with the standard procedure for conducting an LCA, using the R package poLCA.^37^ For each model, the Akaike information criterion (AIC), the Bayesian information criterion (BIC) and the relative entropy was calculated. For the AIC and the BIC, lower values indicate better fit, whereas higher relative entropy values indicate good class separation, with the suggested cut-off value of 0.8.^15,38^ Typically, the performance of a model enhances as more classes are added, up to a point where the optimal statistical solution is achieved, after which the quality starts to decline. Researchers typically keep extending the model by adding one class at a time until the point of deterioration is reached.^15^ For instance, if a model with three classes performs better than one with two, then the model with four classes should be estimated next and compared with the three-class model. Accordingly, after each added class, the AIC and BIC as well as the entropy were examined and compared, as separate values as well as in relation to each other, while also considering the number of classes found in previous research to decide whether another class should be added.

Since the BIC is generally considered the most reliable fit statistic in LCA,^15,39^ it was chosen as the primary measure for model selection. Also considered in the model selection, albeit more collectively, were the AIC, the relative entropy, the number of sample members in each class and conceptual considerations and interpretability.

After identifying the best LCA model, primarily by BIC, each individual was assigned a specific class based on the posterior class membership probabilities. Differences between classes regarding continuous variables were tested using analyses of variance, chi-squared tests were used for categorical variables and Kruskal–Wallis tests were used for ordinal scaled variables. Effect sizes were calculated with eta-squared and Cramer's *V*. All significant tests were additionally tested with pairwise Bonferroni corrected comparisons.

## Results

### Participants

Participants (*N* = 875) were on average 17.72 years old, and 50.4% were female. The majority had Swiss or German nationality, and a variety of different educational qualifications (Table 1). The majority (80.4%; *n* = 569) had an axis 1 disorder, with affective (*n* = 232, 32.9%) and anxiety disorders (*n* = 191, 27.0%) being most prevalent (Table 2). There were 72 transitions to psychosis in the total sample, with a mean time to transition of 14.75 (s.d. = 12.55, median 9.56, minimum 0.36, maximum 50.30) months. In the BEARS-Kid subsample, there were 13 transitions, with a mean time to transition of 13.18 (s.d. = 12.42, median 7.36, minimum 2.30, maximum 38.11) months; and in the FETZ Cologne subsample there were 59 transitions, with a mean time to transition of 15.05 (s.d. = 12.65, median 9.63, minimum 0.36, maximum 50.30) months. There were no transitions in the FETZ Bern subsample (Supplementary Table 7).

**Table 1:** Sociodemographic description of the three latent classes

Sociodemographic description	Total (*N* = 875)	Class 1 (*n* = 523)	Class 2 (*n* = 97)	Class 3 (*n* = 255)	Statistics, effect size	Significant *post hoc* tests, Bonferroni corrected
Age, mean (s.d.)	17.72 (5.38)	16.23 (4.60)	23.59 (5.67)	18.54 (5.04)	*F*(2, 872) = 99.02, *P* < 0.001, η^2^ = 0.19	1 *v.* 2; 1 *v.* 3; 2 *v.* 3
Gender, *n* (%) [standardised residual]					χ^2^(2) = 2.61, *P* = 0.271, *V* = 0.05	
Female	441 (50.4)	261 (49.9)	43 (44.3)	137 (53.7)		
Nationality, *n* (%) [standardised residual]					χ^2^(8) = 124.57, *P* < 0.001, *V* = 0.27	1 *v.* 2; 2 *v.* 3
Switzerland	480 (54.9)	332 (63.5) [6.25]	5 (8.2) [−10.43]	143 (56.1)		
Germany	305 (34.9)	142 (27.2) [−5.83]	79 (81.4) [10.21]	84 (32.9)		
Other	90 (10.2)	49 (9.4)	13 (13.4)	28 (11.0)		
Education (ISCED), *n* (%) [standardised residual]					χ^2^(14) = 46.60, *P* < 0.001, *V* = 0.18	
Early childhood education (ISCED 0)	5 (0.7)	4 (0.8)	0 (0)	1 (0.5)		
Primary education (ISCED 1)	103 (14.4)	95 (19.3) [5.48]	0 (0)	8 (3.8) [−5.25]		
Lower secondary education (ISCED 2)	286 (40.1)	192 (38.9)	5 (55.6)	89 (42.2)		
Apprenticeship (ISCED 3.4)	7 (0.8)	4 (0.8)	0 (0)	3 (1.4)		
High school graduation (ISCED 3.5)	152 (21.3)	100 (20.3)	1 (11.1)	51 (24.2)		
Post-secondary non-tertiary education (ISCED 4)	6 (0.8)	2 (0.4)	1 (11.1)	3 (1.4)		
Short-cycle tertiary education (ISCED 5)	119 (16.7)	73 (14.8) [−2.02]	2 (22.2) [3.39]	44 (20.9)		
Master's or equivalent (ISCED 7)	35 (4.9)	23 (4.7)	0 (0)	12 (5.7)		
Occupation, *n* (%) [standardised residual]					χ^2^(10) = 8.24, *P* = 0.606, *V* = 0.08	
Unemployed	36 (5.0)	24 (4.8)	0 (0)	12 (5.6)		
Protected employment	25 (3.5)	17 (3.4)	0 (0)	8 (3.8)		
Part-time	4 (0.6)	1 (0.2)	0 (0)	3 (1.4)		
Employed	631 (87.9)	441 (88.9)	8 (88.9)	182 (85.4)		
Other	20 (0.3)	12 (2.4)	1 (11.1)	7 (3.3)		
Centre, *n* (%) [standardised residual]					χ^2^(4) = 428.33, *P* < 0.001, *V* = 0.49	1 *v*. 2; 1 *v.* 3; 2 *v.* 3
FETZ Bern	257 (29.4)	151 (28.9)	4 (4.1) [−5.79]	102 (40.0) [4.43]		
BEARS-Kid	461 (52.7)	345 (66.0) [9.59]	5 (5.2) [−9.94]	111 (43.5) [−3.48]		
FETZ Cologne	157 (17.9)	27 (5.2) [−12.01]	88 (90.7) [19.81]	42 (16.5)		

Cramer's *V* interpretation: 0.1 = weak effect, 0.3 = moderate effect, 0.5 = strong effect. Partial η^2^: 0.01 = weak effect, 0.06 = moderate effect, 0.14 = strong effect. The standardised residual is only presented for cells with a value of ≥|1.96|, which equals significant deviation from the expected cell frequency, with positive values indicating higher than expected cell frequency and negative values indicating lower than expected cell frequency. ISCED, International Standard Classification of Education; FETZ, Early Recognition and Intervention Centre for Mental Crisis; BEARS-Kid, Bi-national Evaluation of At-Risk Symptoms in Children and Adolescents.

**Table 2:** Clinical characteristics of the three latent classes

Clinical characteristic	Total (*N* = 875)	Class 1 (*n* = 523)	Class 2 (*n* = 97)	Class 3 (*n* = 255)	Statistics, effect size	Significant *post hoc* tests, Bonferroni corrected
SOFAS, mean (s.d.)	58.01 (13.08)	61.33 (12.15)	44.64 (12.66)	56.26 (11.43)	*F*(2, 867) = 82.20, *P* < 0.001, η^2^ = 0.16	1 *v.* 2; 1 *v.* 3; 2 *v.* 3
Negative symptoms, median (s.d.)						
N1: Social anhedonia	2.00 (1.68)	1.00 (1.49)	4.00 (1.62)	2.00 (1.64)	*H*(2) = 128.47, *P* < 0.001, η^2^ = 0.15	1 *v.* 2; 1 *v.* 3; 2 *v.* 3
N2: Avolition	2.00 (1.59)	2.00 (1.46)	4.00 (1.49)	3.00 (1.34)	*H*(2) = 171.81, *P* < 0.001, η^2^ = 0.20	1 *v.* 2; 1 *v.* 3; 2 *v.* 3
N3: Expression of emotion	0.00 (1.49)	0.00 (1.15)	3.00 (2.04)	1.00 (1.51)	*H*(2) = 94.13, *P* < 0.001, η^2^ = 0.11	1 *v.* 2; 1 *v.* 3; 2 *v.* 3
N4: Experience of emotion and self	1.00 (1.89)	0.00 (1.60)	3.00 (2.02)	3.00 (1.83)	*H*(2) = 146.24, *P* < 0.001, η^2^ = 0.17	1 *v.* 2; 1 *v.* 3
N5: Ideational richness	0.00 (1.15)	0.00 (0.64)	2.00 (1.98)	0.00 (0.94)	*H*(2) = 171.04, *P* < 0.001, η^2^ = 0.19	1 *v.* 2; 1 *v.* 3; 2 *v.* 3
N6: Occupational functioning	2.00 (1.89)	2.00 (1.81)	4.00 (1.59)	3.00 (1.83)	*H*(2) = 92.18, *P* < 0.001, η^2^ = 0.10	1 *v.* 2; 1 *v.* 3; 2 *v.* 3
Disorganised symptoms, median (s.d.)						
D1: Odd behaviour of appearance	0.00 (0.79)	0.00 (0.41)	0.00 (1.63)	0.00 (0.68)	*H*(2) = 127.89, *P* < 0.001, η^2^ = 0.14	1 *v.* 2; 1 *v.* 3; 2 *v.* 3
D2: Bizarre thinking	0.00 (1.56)	0.00 (1.17)	0.00 (1.56)	0.50 (1.87)	*H*(2) = 126.94, *P* < 0.001, η^2^ = 0.14	1 *v.* 2; 1 *v.* 3
D3: Trouble with focus and attention	2.00 (1.38)	2.00 (1.27)	3.00 (1.60)	3.00 (1.21)	*H*(2) = 112.11, *P* < 0.001, η^2^ = 0.13	1 *v.* 2; 1 *v.* 3; 2 *v.* 3
D4: Impairment in personal hygiene	0.00 (0.98)	0.00 (0.70)	1.00 (1.63)	0.00 (0.92)	*H*(2) = 83.23, *P* < 0.001, η^2^ = 0.09	1 *v.* 2; 2 *v.* 3
General symptoms, median (s.d.)						
G1: Sleep disturbance	2.00 (1.54)	2.00 (1.43)	3.00 (1.87)	3.00 (1.28)	*H*(2) = 98.24, *P* < 0.001, η^2^ = 0.11	1 *v.* 2; 1 *v.* 3; 2 *v.* 3
G2: Dysphoric mood	3.00 (1.72)	2.00 (1.62)	4.00 (1.39)	4.00 (1.47)	*H*(2) = 171.18, *P* < 0.001, η^2^ = 0.19	1 *v.* 2; 1 *v.* 3; 2 *v.* 3
G3: Motor disturbances	0.00 (1.24)	0.00 (1.01)	0.00 (1.64)	0.00 (1.35)	*F*(2, 869) = 59.38, *P* < 0.001, η^2^ = 0.06	1 *v.* 2; 1 *v.* 3; 2 *v.* 3
G4: Impaired tolerance to normal stress	2.00 (1.85)	1.00 (1.61)	5.00 (1.76)	2.00 (1.73)	*H*(2) = 160.35, *P* < 0.001, η^2^ = 0.18	1 *v.* 2; 1 *v.* 3; 2 *v.* 3
Neurocognitive deficits, *n* (%) [standardised residual]						
Verbal memory	230 (26.6)	113 (21.9) [−3.84]	39 (40.2) [3.22]	78 (31.1)	χ^2^(2) = 17.73, *P* < 0.001, *V* = 0.14	1 *v.* 2
Processing speed	288 (33.3)	144 (27.8) [−4.16]	50 (52.6) [4.25]	94 (37.2)	χ^2^(2) = 24.75, *P* < 0.001, *V* = 0.17	1 *v.* 2
(Verbal) executive functions	499 (57.2)	278 (53.5) [−2.93]	73 (76.0) [3.90]	148 (58.7)	χ^2^(2) = 17.13, *P* < 0.001, *V* = 0.14	1 *v.* 2; 2 *v.* 3
Spatial memory	133 (15.4)	84 (16.2)	13 (13.5)	36 (14.3)	χ^2^(2) = 0.78, *P* = 0.677, *V* = 0.03	
Axis-1 disorder^a^, *n* (%) [standardised residual]	569 (80.4)	402 (82.4)	8 (88.9)	159 (75.4)	χ^2^(2) = 5.02, *P* = 0.081, *V* = 0.08	
Affective disorders, *n* (%) [standardised residual]	232 (32.9)	110 (22.7) [−8.58]	7 (77.8) [2.88]	115 (54.5) [7.98]	χ^2^(2) = 75.75, *P* < 0.001, *V* = 0.32	1 *v.* 2; 1 *v.* 3
Anxiety disorders, *n* (%) [standardised residual]	191 (27.0)	113 (23.2) [−3.40]	5 (55.6)	73 (24.6) [2.96]	χ^2^(2) = 13.46, *P* = 0.001, *V* = 0.14	1 *v.* 3
Obsessive–compulsive disorder, *n* (%) [standardised residual]	60 (8.5)	47 (9.7)	1 (11.1)	12 (5.7)	χ^2^(2) = 3.00, *P* = 0.224, *V* = 0.06	
Post-traumatic stress disorder^b^, *n* (%) [standardised residual]	10 (1.4)	2 (0.4) [−3.37]	1 (11.1) [2.47]	7 (3.3) [2.80]	χ^2^(2) = 15.10, *P* < 0.001, *V* = 0.14	1 *v.* 3
Somatoform disorders^b^, *n* (%) [standardised residual]	7 (1.0)	5 (1.0)	0 (0)	2 (1.0)	χ^2^(2) = 0.11, *P* = 0.948, *V* = 0.01	
Eating disorders^b^, *n* (%) [standardised residual]	108 (15.3)	96 (19.7) [4.86]	0 (0)	12 (5.7) [−4.60]	χ^2^(2) = 23.84, *P* < 0.001, *V* = 0.18	1 *v.* 3
Substance addiction^b^, *n* (%) [standardised residual]	16 (2.3)	7 (1.4) [−2.20]	1 (11.1)	8 (3.8)	χ^2^(2) = 6.92, *P* = 0.032, *V* = 0.10	
Substance misuse^b^, *n* (%) [standardised residual]	37 (5.2)	19 (3.9) [−2.37]	2 (25.0) [2.52]	16 (7.6)	χ^2^(2) = 10.34, *P* = 0.006, *V* = 0.12	
Transition to psychosis, *n* (%) [standardised residual]	72 (12.2)	5 (1.3) [−10.55]	38 (55.1) [11.56]	29 (19.6) [3.16]	χ^2^(2) = 166.60, *P* < 0.001, *V* = 0.53	1 *v.* 2; 1 *v.* 3; 2 *v.* 3

Cramer's *V* interpretation: 0.1 = weak effect, 0.3 = moderate effect, 0.5 = strong effect; η^2^: 0.01 = weak effect, 0.06 = moderate effect, 0.14 = strong effect. Standardised residual is only presented for cells with a value of ≥|1.96|, which equals significant deviation from the expected cell frequency, with positive values indicating higher than expected cell frequency and negative values indicating lower than expected cell frequency. SOFAS, Social and Occupational Functioning Assessment Scale; MINI/MINI-Kid, Mini-International Neuropsychiatric Interview.

a. Percentages not calculated for total sample, but for the subsample with completed MINI/MINI-Kid (*n* = 718).

b. χ^2^ approximation may be incorrect owing to small number of cases; Bonferroni-corrected critical *P* = 0.016.

### Result of the LCA

**Fig. 1:**
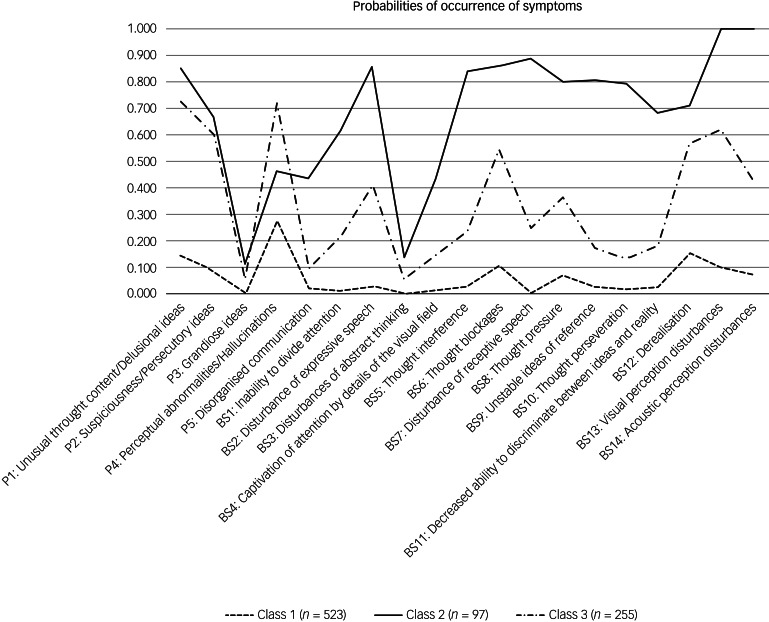
Latent profile plot of clinical high-risk symptoms across the three classes. On the *x*-axis are the basic symptoms and the attenuated positive symptoms. The *y*-axis shows the average probability for the symptom to occur separated for each subgroup.

An overview of the various LCA models tested can be found in Supplementary Table 2. Although the six-class solution showed the lowest AIC (11125.93) and highest entropy (0.974), but only the fourth-lowest BIC (11694.06), the three-class solution had the lowest BIC across the tested models (BIC = 11517.68, AIC = 11236.01 (fourth-lowest), entropy of 0.871 (fourth-highest)). The six-class solution consisted of one class with <5% of the total sample and fewer than 50 individuals, respectively. For this reason, as well as the fact that the highest entropy may not necessarily represent the best-fitting model, but possibly an overfit model,^39^ and for better interpretability, the three-class solution was chosen (Fig. 1).

### Description and comparison of the classes

When the three classes were compared for the severity of the indicator variables (SIPS positive items and basic symptoms) (Fig. 1, Supplementary Tables 3 and 5) and the other clinical and neurocognitive characteristics (Table 2, Supplementary Table 5), the largest class 1, with 532 participants, had low probability of APS/BIPS and basic symptoms, and the lowest median scores on the SIPS positive items (0.00–1.00) and basic symptoms (0.00) (Fig. 1, Supplementary Table 3). In addition, it showed some of the lowest median severity of SIPS negative, disorganised and general symptoms (Table 2). Although there were similar high overall rates of comorbidities across all classes, class 1 included the highest amount of eating disorders, but least amount of affective, anxiety, post-traumatic stress and substance dependence disorders, as well as the lowest amount of participants who transitioned to psychosis. It also had the highest mean SOFAS score that still indicated some difficulties in functioning. Moreover, class 1 had the lowest rates of deficits in verbal memory, processing speed and executive functions, but similar rates of deficits in spatial memory across classes (Table 2). It was termed ‘low symptom load’.

The smallest class 2, with 97 participants, had the highest probability of all APS/BIPS and basic symptoms with the exception of ‘P4: Perceptual abnormalities/hallucinations’, and the highest median scores in all of the CHR symptoms (basic symptoms median: 0.00–4.00, *P* median: 0.00–4.00) except for P4 (Fig. 1, Supplementary Table 3). Similarly, this class had the highest median scores in all negative, disorganised and general items of the SIPS; the lowest mean SOFAS score indicating serious impairments in functioning; slightly increased rates of anxiety, post-traumatic stress and substance misuse disorders, the highest amount of people who transitioned to psychosis and the highest rates of deficits in verbal memory, processing speed and executive functions (Table 2). It was termed ‘high symptom load’.

Relative to the other two groups, the medium-sized class 3 (*n* = 255) had a medium probability for, and medium median severity of, all APS/BIPS and criteria-relevant basic symptoms except for P4 (basic symptoms median: 0.00–2.00, *P* median*:* 0.00–4.00), which had the highest probability of being in APS/BIPS severity and the highest median score of P4 (Fig. 1, Supplementary Table 3). Thus, it was termed ‘predominant hallucinatory experiences’. Similarly, mean scores of the SOFAS, indicating moderate difficulties in functioning, and other SIPS items, were in-between that of the two other classes. However, not all groupwise comparisons were significant, and particularly not when item severity was generally low (Table 2). The same was true for the proportions of those with a neurocognitive deficit that were also intermediate between the two other classes, in particular with respect to a processing speed deficit (Table 2). Moreover, affective, anxiety and post-traumatic stress disorders were increased to a similar degree as in class 2, whereas eating disorders were less prevalent than in class 1; substance misuse and transition to psychosis rates were intermediate to the two other classes (Table 2).

**Fig. 2:**
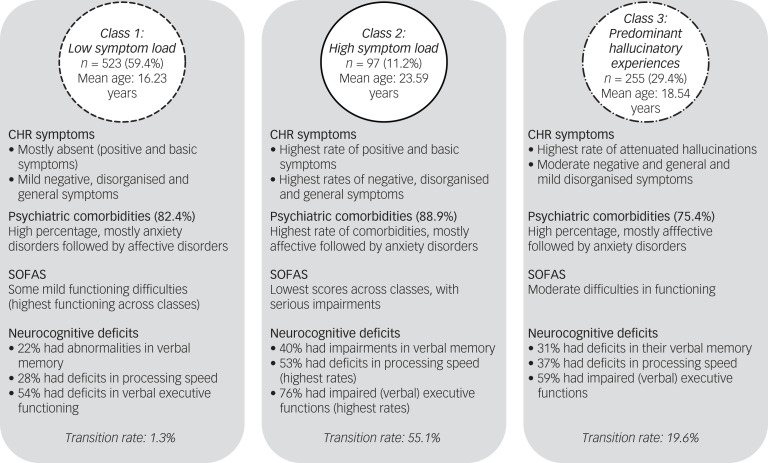
Clinical overview of the three latent classes. CHR, clinical high risk; SOFAS, Social and Occupational Functioning Assessment Scale.

Overall, the symptoms that best differentiated classes were the basic symptoms (η^2^ = 0.08–0.52), whereas neurocognitive deficits did not significantly differentiate classes. Figure 2 gives a summary of the characteristics of the three classes.

With regard to demographic data (Table 1), the mean age of class 1 participants, at 16 years, was significantly lower compared with the other classes, whereas the mean age in class 2, at 23 years, was significantly higher compared with the other two classes. Gender did not distinguish between classes. Furthermore, class 2 was significantly dominated by Germans and included few Swiss; in class 1, this was the other way round (Table 1). Education slightly differed between classes, with slightly lower levels of achievements in the youngest class 1. Occupation, however, was similar across classes that included mostly persons with full-time employment (Table 1).

## Discussion

To our knowledge, this study is the first to explore clinical profiles in patients of specialised early intervention centres for psychosis, based on symptomatic CHR approaches (i.e. basic symptoms and APS), and to comprehensively characterise them using comorbid symptoms, neurocognitive deficits and psychosocial functioning. The best-fitting model comprised three classes characterised by low symptom load (class 1), high symptom load (class 2) and predominant hallucinatory experiences (class 3). These classes showed clinically meaningful differences and, given replication in other samples, may represent more homogenous subgroups for clinical identification and utility.

### CHR classes in the context of existing research

Our study found significant differences in the expression of CHR symptoms across the three classes, with some exceptions of rarely occurring and less pronounced symptoms (i.e. ‘P3: Grandiose ideas’), where the general low variance made finding a significant difference difficult. Measured by the effect sizes, the three classes in the present study were best differentiated by basic symptoms, with the symptom ‘BS7: Disturbances of receptive speech’ showing the largest effect (η^2^ = 0.52), reflecting the relevance of basic symptoms, aligning with previous research.^3^ As shown in a longitudinal study, the number of basic symptoms in UHR individuals significantly increased the risk of transition to psychosis, and this is likely to be mediated mainly by the significantly higher frequency of disturbances of receptive speech.^40^ Accordingly, and at least partially in accordance with the EPA guidance, emphasising the psychosis-predictive value of BIPS criteria,^3^ we found the most fulfilled BIPS criteria (Supplementary Table 4) in class 2 (Fig. 1, Supplementary Table 3). Additionally, we found the most frequent and strongest disturbances of receptive speech in class 2 (Fig. 1, Supplementary Table 3). Both of these aspects could explain the high transition rate in class 2. Further, the high transition rate in class 2 can also be explained by previous research, which showed a higher probability of transition to psychosis with lower level of functioning (i.e. SOFAS scores),^41^ potentially indicating the presence of underlying neurodevelopmental issues. Although there has been an increased focus on transition rates in research, our study showed (in line with others^7^) that most individuals do not transition to psychosis. Especially in younger samples, one of the factors that could contribute to this is that, depending on age, certain symptoms such as P4 might have a lower disease value.^42,43^ Consistently, the respective symptom in our study is clearly dominant within the youngest class and most common in the second youngest. This aligns with reported higher prevalence and lower clinical significance of perceptual abnormalities/hallucinations in younger samples,^24,44^ and with a lower relation to other positive symptoms.^43^ The latter is also supported by the significantly different transition rates across classes. Moreover, the reported association with lower age and lower transition rates is supported.^3^

Already described in previous research, and evident in our study, is that comorbid disorders in CHR individuals are the norm rather than the exception. Compared with a recent meta-analysis of comorbidities in CHR individuals,^45^ especially class 2 and 3, which contain primarily CHR patients (Supplementary Table 4), have similar prevalence and distributions, especially low numbers of somatoform and eating disorders (Table 2). Additionally, particularly high rates of anxiety disorders and substance misuse in class 2 fit the increased prevalence of anxiety disorders and alcohol misuse in CHR populations compared with clinical controls.^44^ However, the negative correlation of affective disorders as well as of two anxiety disorders with a transition found in the meta-analysis^44^ does not fit with the high prevalence in class 2 with the highest transition rate. Yet, since depressive and anxiety disorders are the most frequently occurring comorbid disorders (as in first-episode psychosis),^46^ and also exhibits symptoms that most frequently lead to help-seeking,^47^ this contradiction probably indicates that both disorders may have no direct specific connection with a transition risk, but that the correlations may be mediated in each case by other variables (such as level of functioning). Similarly, the distribution of functional levels fits well with similar comorbidity rates, which again underlines the relevance of CHR symptoms.^48^

### General implications and neurocognitive deficits in the scope of clinical care

The identification of homogenous subgroups in our study allows for enhanced clinical utility, particularly regarding targeted intervention. Given the high rates of psychiatric comorbidity in all classes, addressing anxiety and affective symptoms, in particular, is indicated. Additionally, although class 1 had a lack of CHR symptoms, negative symptoms in this group may be ‘secondary’ (i.e. not necessarily related to CHR symptoms themselves, but rather, part of psychiatric or medical comorbidities, adverse effects of treatment or environmental factors), and may be best reduced by treating other symptoms, including anxiety. However, if negative symptoms do not respond to treatment, persist over time and interfere with normal role functioning, they should be carefully managed and monitored by clinicians, given that they have been consistently linked to poor outcomes.^49^

Because class 2 included the highest general psychopathology, both a broad approach attending to general symptom severity and prioritising targeted modular interventions for basic symptoms and attenuated positive symptoms (owing to their distressing nature) may be indicated.^50^ Intervention for broader (psychosocial) functioning should also include attending to negative symptoms, including psychoeducation, behavioural activation, coping, etc. Moreover, close collaboration with workplaces and schools of patients may prevent negative cascading effects (i.e. job loss) by, for example, ensuring increased time allowances for work completion. Further, although negative symptoms are clinically relevant particularly because of their severity and number in the context of class 2, currently, limited effective and evidence-based interventions are available for the treatment of negative symptoms, indicating the critical need for further research.^51^

Interventions specifically tailored to delusional ideas, perceptual symptoms and derealisation would be most relevant for class 3. These likely include modified cognitive–behavioural therapy (CBT) interventions (i.e. CBT for voices),^52^ as well as specialised treatment for derealisation (i.e. group therapy specific to this symptom).^53^ Other emerging self-guided CBT interventions for auditory hallucinations have shown promising results in reducing the effects of voices, as well as other relevant clinical outcomes such as well-being, anxiety and self-esteem.^52^

All classes in our study exhibited some neurocognitive deficits (i.e. in processing speed and (verbal) executive functions) even in the low-risk class, partly aligning with previous research.^18^ However, neurocognitive deficits did not significantly differentiate between classes. To date, few effective pharmacological or psychological interventions are available to ameliorate neurocognitive deficits in CHR individuals, despite neurocognitive deficits having been consistently reported in CHR and psychosis populations.^54^ For groups where neurocognitive disturbances might be related to other comorbid disorders (i.e. anxiety in class 1), treating comorbid symptoms might lead to some improvement in the neurocognitive domains, since anxiety symptoms (among other factors) have been shown to mediate cognitive test performance.^55^ However, in the meantime, the achievement of developmental tasks of young people might be threatened by the potential negative effects of neurocognitive deficits in relation to school education. Therefore, close collaboration between therapists and teachers may be required to adapt to the young people's needs and provide support, where necessary, to enable them to participate successfully in class.

Alternatively, results from emerging interventions including cognitive enhancement therapy in individuals with schizophrenia (e.g. overall improvement in cognition, and beneficial effects on attention/vigilance),^56^ if appropriately adapted, might translate as a promising option for CHR individuals with some neurocognitive deficits – this likely being particularly relevant for class 2 in our study. New opportunities might also be offered by using virtual reality technology for the development of innovative neuropsychological intervention tools.^57^ In CHR individuals, virtual reality environments might offer the opportunity to improve not only the specific neurocognitive deficits with which they present (i.e. processing speed, verbal memory), but also for the treatment of psychotic symptoms,^58^ or by combining virtual reality with cognitive remediation programmes,^59^ depending on the respective financial resources.

### Strengths and limitations

A clear strength of this study is the large sample size, comprising a wide age range of patients, the comprehensive assessment of CHR symptoms, including basic symptoms, and their associations with neurocognitive measures. Further, assessments were clinical interviews conducted by trained psychologists, adding to the validity of the data. However, some limitations should also be acknowledged. First, we did not control for ongoing psychotherapeutic or pharmacological treatment, which might affect several target variables. Further, the use of mainly cross-sectional data aside from information regarding transition to psychosis prevents drawing any conclusions about course of the subgroups regarding other outcomes. Additionally, data is from three different centres, and the possibility of centre effects cannot entirely be excluded (see Supplementary Table 6 for a description of the sample across centres). Finally, lack of a validation in an external sample is another limitation.

### Implications for the future

The utility of basic symptoms alongside with APS to distinguish between classes in our study highlights not only the need for assessment of these symptoms but also their potential to help further understand the complexity of CHR presentation, supporting its place in CHR diagnosis according to the EPA guidance.^3^ Despite some challenges associated with the assessment of basic symptoms (given its subjectivity) and its distal relationship to fully manifesting psychosis (being the very earliest subjectively experienced symptoms of psychotic development), it co-occurs with functional difficulties and, ultimately, psychological distress. Therefore, basic symptoms may gain new relevance for broader conceptualisation of CHR symptoms not limited to transition, but rather individual clinical presentation associated with psychological distress. Moreover, given the rarity of specific treatments available for basic symptoms (aside from derealisation), our findings call for increased attention to the development of effective interventions for these distressing symptoms.

Our study is among the first to explore clinical profiles in CHR samples beyond the explicit notion of predicting transition to psychosis, focusing on broader clinical presentation (including functioning) targetable by modular intervention. This adds to the broader research and clinical aim of parsing out heterogeneity in this clinical group and, consequently, aiding in the assessment, identification and intervention of help-seeking individuals.

## Supporting information

Stüble et al. supplementary material 1 Stüble et al. supplementary material

Stüble et al. supplementary material 2 Stüble et al. supplementary material

## Data Availability

Data will be made available on reasonable request to the corresponding author, C.M.

## References

[1] Csillag C, Nordentoft M, Mizuno M, Jones PB, Killackey E, Taylor M, et al. Early intervention services in psychosis: from evidence to wide implementation. Early Interv Psychiatry 2016; 10(6): 540–6. 26362703 10.1111/eip.12279

[2] Fusar-Poli P, Borgwardt S, Bechdolf A, Addington J, Riecher-Rössler A, Schultze-Lutter F, et al. The psychosis high-risk state. JAMA Psychiatry 2013; 70(1): 107–20. 23165428 10.1001/jamapsychiatry.2013.269 PMC4356506

[3] Schultze-Lutter F, Michel C, Schmidt SJ, Schimmelmann BG, Maric NP, Salokangas RKR, et al. EPA guidance on the early detection of clinical high risk states of psychoses. Eur Psychiatry 2015; 30(3): 405–16. 25735810 10.1016/j.eurpsy.2015.01.010

[4] Schultze-Lutter F, Debbané M, Theodoridou A, Wood SJ, Raballo A, Michel C, et al. Revisiting the basic symptom concept: toward translating risk symptoms for psychosis into neurobiological targets. Front Psychiatry 2016; 7: 9. 10.3389/fpsyt.2016.00009 PMC4729935 26858660

[5] Fusar-Poli P, Cappucciati M, Borgwardt S, Woods SW, Addington J, Nelson B, et al. Heterogeneity of psychosis risk within individuals at clinical high risk: a meta-analytical stratification. JAMA Psychiatry 2016; 73(2): 113. 26719911 10.1001/jamapsychiatry.2015.2324

[6] Beck K, Studerus E, Andreou C, Egloff L, Leanza L, Simon AE, et al. Clinical and functional ultra-long-term outcome of patients with a clinical high risk (CHR) for psychosis. Eur Psychiatry 2019; 62: 30–7. 31514058 10.1016/j.eurpsy.2019.08.005

[7] Michel C, Ruhrmann S, Schimmelmann BG, Klosterkötter J, Schultze-Lutter F. Course of clinical high-risk states for psychosis beyond conversion. Eur Arch Psychiatry Clin Neurosci 2018; 268(1): 39–48. 28054132 10.1007/s00406-016-0764-8

[8] Healy C, Brannigan R, Dooley N, Coughlan H, Clarke M, Kelleher I, et al. Childhood and adolescent psychotic experiences and risk of mental disorder: a systematic review and meta-analysis. Psychol Med 2019; 49(10): 1589–99. 31088578 10.1017/S0033291719000485

[9] Salazar de Pablo G, Soardo L, Cabras A, Pereira J, Kaur S, Besana F, et al. Clinical outcomes in individuals at clinical high risk of psychosis who do not transition to psychosis: a meta-analysis. Epidemiol Psychiatr Sci 2022; 31: e9. 35042573 10.1017/S2045796021000639 PMC8786617

[10] Hartmann JA, Schmidt SJ, McGorry PD, Berger M, Berger GE, Chen EYH, et al. Trajectories of symptom severity and functioning over a three-year period in a psychosis high-risk sample: a secondary analysis of the neurapro trial. Behav Res Ther 2020; 124: 103527. 10.1016/j.brat.2019.103527 31790853

[11] Feczko E, Miranda-Dominguez O, Marr M, Graham AM, Nigg JT, Fair DA. The heterogeneity problem: approaches to identify psychiatric subtypes. Trends Cogn Sci 2019; 23(7): 584–601. 31153774 10.1016/j.tics.2019.03.009 PMC6821457

[12] Carpenter WT. Clinical high risk controversies and challenge for the experts. Schizophr Bull 2018; 44(2): 223–5. 29272521 10.1093/schbul/sbx182 PMC5815099

[13] Mittal VA, Addington JM. Embracing heterogeneity creates new opportunities for understanding and treating those at clinical-high risk for psychosis. Schizophr Res 2021; 227: 1–3. 33288356 10.1016/j.schres.2020.11.015

[14] Kendler KS, Karkowski LM, Walsh D. The structure of psychosis: latent class analysis of probands from the roscommon family study. Arch Gen Psychiatry 1998; 55(6): 492–9. 9633666 10.1001/archpsyc.55.6.492

[15] Weller BE, Bowen NK, Faubert SJ. Latent class analysis: a guide to best practice. J Black Psychol 2020; 46(4): 287–311.

[16] Ryan AT, Addington J, Bearden CE, Cadenhead KS, Cornblatt BA, Mathalon DH, et al. Latent class cluster analysis of symptom ratings identifies distinct subgroups within the clinical high risk for psychosis syndrome. Schizophr Res 2018; 197: 522–30. 29279247 10.1016/j.schres.2017.12.001 PMC6015526

[17] Valmaggia LR, Stahl D, Yung AR, Nelson B, Fusar-Poli P, McGorry PD, et al. Negative psychotic symptoms and impaired role functioning predict transition outcomes in the at-risk mental state: a latent class cluster analysis study. Psychol Med 2013; 43(11): 2311–25. 23442767 10.1017/S0033291713000251

[18] Catalan A, Salazar de Pablo G, Aymerich C, Damiani S, Sordi V, Radua J, et al. Neurocognitive functioning in individuals at clinical high risk for psychosis: a systematic review and meta-analysis. JAMA Psychiatry 2021; 78(8): 859–67. 34132736 10.1001/jamapsychiatry.2021.1290 PMC8209603

[19] De Herdt A, Wampers M, Vancampfort D, De Hert M, Vanhees L, Demunter H, et al. Neurocognition in clinical high risk young adults who did or did not convert to a first schizophrenic psychosis: a meta-analysis. Schizophr Res 2013; 149(1–3): 48–55. 23830855 10.1016/j.schres.2013.06.017

[20] Schultze-Lutter F, Theodoridou A. The concept of basic symptoms: its scientific and clinical relevance. World Psychiatry 2017; 16(1): 104–5. 28127912 10.1002/wps.20404 PMC5269478

[21] Michel C, Kaess M, Flückiger R, Büetiger JR, Schultze-Lutter F, Schimmelmann BG, et al. The Bern early recognition and intervention centre for mental crisis (FETZ Bern)-An 8-year evaluation. Early Interv Psychiatry 2022; 16(3): 289–301. 33960114 10.1111/eip.13160

[22] Schultze-Lutter F, Picker H, Ruhrmann S, Klosterkötter J. [The Cologne early recognition and intervention center for mental crises (FETZ). evaluation of service use]. Med Klin Munich Ger 1983 2008; 103(2): 81–9. 10.1007/s00063-008-1012-4 18270664

[23] Schimmelmann BG, Michel C, Martz-Irngartinger A, Linder C, Schultze-Lutter F. Age matters in the prevalence and clinical significance of ultra-high-risk for psychosis symptoms and criteria in the general population: findings from the BEAR and BEARS-kid studies. World Psychiatry 2015; 14(2): 189–97. 26043337 10.1002/wps.20216 PMC4471976

[24] Schultze-Lutter F, Ruhrmann S, Michel C, Kindler J, Schimmelmann BG, Schmidt SJ. Age effects on basic symptoms in the community: a route to gain new insight into the neurodevelopment of psychosis? Eur Arch Psychiatry Clin Neurosci 2020; 270(3): 311–24. 30361925 10.1007/s00406-018-0949-4 PMC7069926

[25] Schultze-Lutter F, Walger P, Franscini M, Traber-Walker N, Osman N, Walger H, et al. Clinical high-risk criteria of psychosis in 8–17-year-old community subjects and inpatients not suspected of developing psychosis. World J Psychiatry 2022; 12(3): 425–49. 35433326 10.5498/wjp.v12.i3.425 PMC8968502

[26] McGlashan T, Walsh B, Woods S. The Psychosis-Risk Syndrome: Handbook for Diagnosis and Follow-up. Oxford University Press, 2010.

[27] Schultze-Lutter F, Addington J, Ruhrmann S, Klosterkötter J. Schizophrenia Proneness Instrument, Adult Version (SPI-A). Rome Giovanni Fioriti, 2007.

[28] Schultze-Lutter F, Koch E. Schizophrenia Proneness Instrument: Child and Youth Version (SPI-CY). Fioriti Rome, 2010. 10.1016/j.schres.2013.02.014 23473813

[29] Schultze-Lutter F, Ruhrmann S, Fusar-Poli P, Bechdolf A, Schimmelmann BG, Klosterkotter J. Basic symptoms and the prediction of first-episode psychosis. Curr Pharm Des 2012; 18(4): 351–7. 22239566 10.2174/138161212799316064

[30] Sheehan DV, Lecrubier Y, Sheehan KH, Amorim P, Janavs J, Weiller E, et al. The Mini-international neuropsychiatric interview (MINI): the development and validation of a structured diagnostic psychiatric interview for DSM-IV and ICD-10. J Clin Psychiatry 1998; 59(20): 22–33. 9881538

[31] American Psychiatric Association. Diagnostic and Statistical Manual of Mental Disorders, Fourth Edition, Text Revision (DSM-IV-TR®). American Psychiatric Association, 2010.

[32] American Psychiatric Association. Diagnostic and Statistical Manual of Mental Disorders: DSM-IV. Vol. 4. American Psychiatric Association, 1994.

[33] Hilsenroth MJ, Ackerman SJ, Blagys MD, Baumann BD, Baity MR, Smith SR, et al. Reliability and validity of DSM-IV axis V. Am J Psychiatry 2000; 157(11): 1858–63. 11058486 10.1176/appi.ajp.157.11.1858

[34] Rybarczyk B. Social and occupational functioning assessment scale (SOFAS). In Encyclopedia of Clinical Neuropsychology (eds JS Kreutzer, J DeLuca, B Caplan): 2313. Springer, 2011.

[35] Morosini PL, Magliano L, Brambilla L, Ugolini S, Pioli R. Development, reliability and acceptability of a new version of the DSM- IV social occupational functioning assessment scale (SOFAS) to assess routine social functioning. Acta Psychiatr Scand 2000; 101(4): 323–9. 10782554

[36] Michel C, Ruhrmann S, Schimmelmann BG, Klosterkötter J, Schultze-Lutter F. A stratified model for psychosis prediction in clinical practice. Schizophr Bull 2014; 40(6): 1533–42. 24609300 10.1093/schbul/sbu025 PMC4193710

[37] Linzer DA, Lewis JB. poLCA: an *R* package for polytomous variable latent class analysis. J Stat Softw 2011; 42(10): 1–29.

[38] Muthén BO. *Re: What Is a Good Value of Entropy?.* Mplus, 2008 (https://www.statmodel.com/discussion/messages/13/2562.html).

[39] Sinha P, Calfee CS, Delucchi KL. Practitioner's guide to latent class analysis: methodological considerations and common pitfalls. Crit Care Med 2021; 49(1): e63–79. 33165028 10.1097/CCM.0000000000004710 PMC7746621

[40] Bang M, Park JY, Kim KR, Lee SY, Song YY, Kang JI, et al. Psychotic conversion of individuals at ultra-high risk for psychosis: the potential roles of schizotypy and basic symptoms. Early Interv Psychiatry 2019; 13(3): 546–54. 29218852 10.1111/eip.12518

[41] Cornblatt BA, Carrión RE, Addington J, Seidman L, Walker EF, Cannon TD, et al. Risk factors for psychosis: impaired social and role functioning. Schizophr Bull 2012; 38(6): 1247–57. 22080497 10.1093/schbul/sbr136 PMC3494064

[42] Schultze-Lutter F, Kindler J, Ambarini TK, Michel C. Positive psychotic symptoms in childhood and adolescence. Curr Opin Psychol 2022; 45: 101287. 35016089 10.1016/j.copsyc.2021.11.007

[43] Jimeno N, Gomez-Pilar J, Poza J, Hornero R, Vogeley K, Meisenzahl E, et al. (Attenuated) hallucinations join basic symptoms in a transdiagnostic network cluster analysis. Schizophr Res 2022; 243: 43–54. 35231833 10.1016/j.schres.2022.02.018

[44] Schultze-Lutter F, Schimmelmann BG, Flückiger R, Michel C. Effects of age and sex on clinical high-risk for psychosis in the community. World J Psychiatry 2020; 10(5): 101–24. 32477906 10.5498/wjp.v10.i5.101 PMC7243619

[45] Solmi M, Soardo L, Kaur S, Azis M, Cabras A, Censori M, et al. Meta-analytic prevalence of comorbid mental disorders in individuals at clinical high risk of psychosis: the case for transdiagnostic assessment. Mol Psychiatry 2023; 28: 2291–300. 37296309 10.1038/s41380-023-02029-8 PMC10611568

[46] Wilson RS, Yung AR, Morrison AP. Comorbidity rates of depression and anxiety in first episode psychosis: a systematic review and meta-analysis. Schizophr Res 2020; 216: 322–9. 31791816 10.1016/j.schres.2019.11.035

[47] Schultze-Lutter F, Michel C, Ruhrmann S, Schimmelmann BG. Prevalence and clinical significance of DSM-5–attenuated psychosis syndrome in adolescents and young adults in the general population: the Bern epidemiological at-risk (BEAR) study. Schizophr Bull 2014; 40(6): 1499–508. 24353096 10.1093/schbul/sbt171 PMC4193691

[48] Schultze-Lutter F, Michel C, Ruhrmann S, Schimmelmann BG. Prevalence and clinical relevance of interview-assessed psychosis-risk symptoms in the young adult community. Psychol Med 2018; 48(7): 1167–78. 28889802 10.1017/S0033291717002586 PMC6088777

[49] Devoe DJ, Braun A, Seredynski T, Addington J. Negative symptoms and functioning in youth at risk of psychosis: a systematic review and meta-analysis. Harv Rev Psychiatry 2020; 28(6): 341–55. 33156155 10.1097/HRP.0000000000000273 PMC8527275

[50] Schmidt SJ, Hurlemann R, Schultz J, Wasserthal S, Kloss C, Maier W, et al. Multimodal prevention of first psychotic episode through N-acetyl-l-cysteine and integrated preventive psychological intervention in individuals clinically at high risk for psychosis: protocol of a randomized, placebo-controlled, parallel-group trial. Early Interv Psychiatry 2019; 13(6): 1404–15. 30784233 10.1111/eip.12781

[51] Salazar de Pablo G, Catalan A, Vaquerizo Serrano J, Pedruzo B, Alameda L, Sandroni V, et al. Negative symptoms in children and adolescents with early-onset psychosis and at clinical high-risk for psychosis: systematic review and meta-analysis. Br J Psychiatry 2023; 223(1): 282–94. 37194556 10.1192/bjp.2022.203 PMC10331322

[52] Hazell CM, Hayward M, Cavanagh K, Jones AM, Strauss C. Guided self-help cognitive-behaviour intervention for VoicEs (GiVE): results from a pilot randomised controlled trial in a transdiagnostic sample. Schizophr Res 2018; 195: 441–7. 29033279 10.1016/j.schres.2017.10.004

[53] Flückiger R, Schmidt SJ, Michel C, Kindler J, Kaess M. Introducing a group therapy program (PLAN D) for young outpatients with derealization and depersonalization: a pilot study. Psychopathology 2021; 55(1): 62–8. 34818653 10.1159/000520008

[54] McCleery A, Nuechterlein KH. Cognitive impairment in psychotic illness: prevalence, profile of impairment, developmental course, and treatment considerations. Dialogues Clin Neurosci 2019; 21(3): 239–48. 31749648 10.31887/DCNS.2019.21.3/amccleery PMC6829172

[55] Moritz S, Silverstein SM, Beblo T, Özaslan Z, Zink M, Gallinat J. Much of the neurocognitive impairment in schizophrenia is Due to factors other than schizophrenia itself: implications for research and treatment. Schizophr Bull Open 2021; 2(1): sgaa034.

[56] Wojtalik JA, Mesholam-Gately RI, Hogarty SS, Greenwald DP, Litschge MY, Sandoval LR, et al. Confirmatory efficacy of cognitive enhancement therapy for early schizophrenia: results from a multisite randomized trial. Psychiatr Serv 2022; 73(5): 501–9. 34470506 10.1176/appi.ps.202000552 PMC8888780

[57] Rizzo AA, Schultheis M, Kerns KA, Mateer C. Analysis of assets for virtual reality applications in neuropsychology. Neuropsychol Rehabil 2004; 14(1–2): 207–39.

[58] Bisso E, Signorelli MS, Milazzo M, Maglia M, Polosa R, Aguglia E, et al. Immersive virtual reality applications in schizophrenia Spectrum therapy: a systematic review. Int J Environ Res Public Health 2020; 17(17): 6111. 32842579 10.3390/ijerph17176111 PMC7504018

[59] Perra A, Riccardo CL, De Lorenzo V, De Marco E, Di Natale L, Kurotschka PK, et al. Fully immersive virtual reality-based cognitive remediation for adults with psychosocial disabilities: a systematic scoping review of methods intervention gaps and meta-analysis of published effectiveness studies. Int J Environ Res Public Health 2023; 20(2): 1527. 36674283 10.3390/ijerph20021527 PMC9864668

