# Supplementary material for: Clinical and neurocognitive profiles of a combined clinical high risk for psychosis and clinical control sample: latent class analysis
Source: BJPsych Open. 2024 Dec 5;10(6):e226. doi: 10.1192/bjo.2024.815 (PMC11698148; doi:10.1192/bjo.2024.815)
Supplement: Stüble et al. supplementary material 1 — Stüble et al. supplementary material [file S2056472424008159sup001.docx]

**Supplementary Material to “Clinical and Neurocognitive Profiles of a Combined Clinical High Risk for Psychosis and Clinical Control Sample: A Latent Class Analysis” (Stüble et al.)**

**Supplemental Table 1. Neurocognitive tests**

| Neuropsychological domain | Neuropsychological tests |
| --- | --- |
| Verbal memory | The Auditory Verbal Learning Test (AVLT)^53,54^ |
| Processing speed | The digit symbol test (DST)^55^ and trail-making test (TMT A)^56^ |
| (Verbal) executive functions | Verbal fluency task (RWT)^57^ and the trail-making test (TMT B)^56^ |
| Spatial memory | Subject Ordered Pointing Task (SOPT)^58,59^ |

*Note.* German versions of all tests have been used.

**Supplemental Table 2. Evaluating Class Solutions**

|  | Model fit criteria | | Diagnostic criteria |
| --- | --- | --- | --- |
| Model | AIC | BIC | Entropy |
| 1 Class | 13873.38 | 13964.09 | - |
| 2 Class | 11555.54 | 11741.73 | 0.901 |
| **3 Class** | **11236.01** | **11517.68** | **0.871** |
| 4 Class | 11203.14 | 11580.30 | 0.878 |
| 5 Class | 11141.71 | 11614.36 | 0.804 |
| 6 Class | 11125.93 | 11694.06 | 0.974 |

*Note: N*=875. AIC: Akaike information criterion; BIC: Bayesian information criterion. Items in bold represent fit indices of the chosen model.

**Supplemental Table 3. Description of indicator variables for LCA**

| Variable, Mdn (SD) | Total  (N = 875) | Class 1  (n=523) | Class 2  (n=97) | Class 3  (n=255) | Statistics, effect size | Significant post-hoc tests, Bonferroni corrected |
| --- | --- | --- | --- | --- | --- | --- |
| Positive symptoms |  |  |  |  |  |  |
| P1: Unusual Thought Content/Delusional Ideas | 2.00 (1.61) | 1.00 (1.24) | 4.00 (1.33) | 3.00 (1.25) | *H*(2)=349.10, *p*<0.001, η^2^=0.40 | 1 vs. 2; 1 vs. 3 |
| P2: Suspiciousness/ Persecutory Ideas | 1.00 (1.64) | 0.00 (1.15) | 3.00 (1.40) | 3.00 (1.59) | *H*(2)=316.60, *p*<0.001, η^2^=0.36 | 1 vs. 2; 1 vs. 3 |
| P3: Grandiose Ideas | 0.00 (0.76) | 0.00 (0.50) | 0.00 (1.09) | 0.00 (0.98) | *H*(2)=22.67, *p* <0.001, η^2^=0.02 | 1 vs. 3 |
| P4: Perceptual Abnormalities/ Hallucinations | 2.00 (1.82) | 1.00 (1.59) | 2.00 (1.57) | 4.00 (1.57) | *H*(2)=212.76, *p*<0.001, η^2^=0.24 | 1 vs. 3; 2 vs. 3; 2 vs. 3 |
| P5: Disorganized Communication | 0.00 (1.18) | 0.00 (0.68) | 2.00 (1.54) | 0.00 (1.22) | *H*(2)=242.99, *p*<0.001, η^2^=0.28 | 1 vs. 2; 1 vs. 3; 2 vs. 3 |
| Basic symptoms |  |  |  |  |  |  |
| BS1: Inability to divide attention | 0.00 (1.54) | 0.00 (0.39) | 3.00 (2.23) | 0.00 (2.00) | *H*(2)= 247.35, *p*<0.001, η^2^=0.28 | 1 vs. 2; 1 vs. 3; 2 vs. 3 |
| BS2: Disturbance of expressive speech | 0.00 (1.69) | 0.00 (0.65) | 3.00 (1.72) | 0.00 (2.08) | *H*(2)= 361.71, *p*<0.001,  η^2^ =0.41 | 1 vs. 2; 1 vs. 3; 2 vs. 3 |
| BS3: Disturbances of abstract thinking | 0.00 (0.45) | 0.00 (0.11) | 0.00 (0.83) | 0.00 (0.75) | *H*(2)= 24.96, *p*<0.001, η^2^=0.26 | 1 vs. 3; 1 vs. 3 |
| BS4: Captivation of attention by details of the visual field | 0.00 (0.83) | 0.00 (0.40) | 0.00 (1.24) | 0.00 (1.32) | *H*(2)= 70.37, *p*<0.001, η^2^=0.08 | 1 vs. 2; 1 vs. 3 |
| BS5: Thought interference | 0.67 (1.55) | 0.00 (0.55) | 4.00 (1.81) | 0.00 (1.77) | *H*(2)= 377.62, *p*<0.001, η^2^=0.43 | 1 vs. 2; 1 vs. 3; 2 vs. 3 |
| BS6: Thought blockages | 0.00 (1.96) | 0.00 (1.00) | 3.00 (1.75) | 2.00 (2.27) | *H*(2)= 269.28, *p*<0.001, η^2^=0.30 | 1 vs. 2; 1 vs. 3; 2 vs. 3 |
| BS7: Disturbance of receptive speech | 0.00 (1.48) | 0.00 (0.22) | 4.00 (1.75) | 0.00 (1.64) | *H*(2)= 455.90, *p*<0.001, η^2^=0.52 | 1 vs. 2; 1 vs. 3; 2 vs. 3 |
| BS8: Thought pressure | 0.00 (1.69) | 0.00 (0.89) | 3.00 (1.89) | 0.00 (1.99) | *H*(2)= 274.55, *p*<0.001, η^2^=0.31 | 1 vs. 2; 1 vs. 3; 2 vs. 3 |
| BS9: Unstable ideas of reference | 0.00 (1.56) | 0.00 (0.53) | 4.00 (1.98) | 0.00 (1.72) | *H*(2)= 375.53, *p*<0.001, η^2^=0.43 | 1 vs. 2; 1 vs. 3; 2 vs. 3 |
| BS10: Thought perseveration | 0.00 (1.32) | 0.00 (0.38) | 3.00 (1.96) | 0.00 (1.29) | *H*(2)= 414.98, *p*<0.001, η^2^=0.47 | 1 vs. 2; 1 vs. 3; 2 vs. 3 |
| BS11: Decreased ability to discriminate between ideas and reality | 0.00 (0.98) | 0.00 (0.46) | 3.00 (1.93) | 0.00 (1.45) | *H*(2)= 118.17, *p*<0.001, η^2^=0.13 | 1 vs. 2; 1 vs. 3; 2 vs. 3 |
| BS12: Derealization | 0.00 (2.07) | 0.00 (1.57) | 3.00 (2.17) | 2.00 (2.44) | *H*(2)= 156.35, *p*<0.001, η^2^=0.18 | 1 vs. 2; 1 vs. 3 |
| BS13: Visual perception disturbances | 0.00 (1.85) | 0.00 (1.00) | 3.00 (1.24) | 2.00 (2.27) | *H*(2)= 320.45, *p*<0.001, η^2^=0.37 | 1 vs. 2; 1 vs. 3 |
| BS14: Acoustic perception disturbances | 0.00 (1.58) | 0.00 (0.81) | 3.00 (1.28) | 0.00 (2.01) | *H*(2)= 280.36, *p*<0.001, η^2^=0.32 | 1 vs. 2; 1 vs. 3; 2 vs. 3 |

*Note:* η2: 0.01=weak effect; 0.06=moderate effect; 0.14=strong effect. Bonferroni corrected critical *p* = 0.016

**Supplemental Table 4. Fulfilled CHR criteria for each class**

|  | Total  (N = 875) | Class 1  (n=532) | Class 2  (n=97) | Class 3  (n=255) |
| --- | --- | --- | --- | --- |
| Risk type, n (%) |  |  |  |  |
| COPER | 332 (66.0) | 90 (53.6) | 71 (73.2) | 171 (71.8) |
| COGDIS | 38 (7.6) | 6 (3.6) | 2 (2.1) | 30 (12.6) |
| APS | 95 (18.9) | 63 (37.5) | 3 (3.1) | 29 (12.2) |
| BIPS | 31 (6.2) | 4 (2.4) | 20 (20.6) | 7 (2.9) |
| GRFD | 7 (1.4) | 5 (3.0) | 1 (1.0) | 1 (0.4) |
| Total | 503 (100) | 168 (100) | 97 (100) | 238 (100) |

*Note.* percentages not calculated for total sample but for individuals fulfilling any risk type; an individual can fulfil one and/or more criteria at the same time

**Supplemental Table 5. P-values for significant group differences (pairwise, Bonferroni corrected with critical *p* = 0.01666667)**

|  | **Class** | **1 vs. 2** | **1 vs. 3** | **2 vs. 3** |
| --- | --- | --- | --- | --- |
| Age |  | *p* < 0.001 | *p* < 0.001 | *p* < 0.001 |
| Nationality |  | *p* < 0.001 | 0.599 | *p* < 0.001 |
| Education^a^ |  | *-* | *-* | - |
| Center |  | *p* < 0.001 | *p* < 0.001 | *p* < 0.001 |
| SOFAS |  | *p* < 0.001 | *p* < 0.001 | *p* < 0.001 |
| P1 |  | *p* < 0.001 | *p* < 0.001 | 0.015 |
| P2 |  | *p* < 0.001 | *p* < 0.001 | 1.000 |
| P3 |  | 0.023 | *p* < 0.001 | 1.000 |
| P4 |  | *p* < 0.001 | *p* < 0.001 | *p* < 0.001 |
| P5 |  | *p* < 0.001 | *p* < 0.001 | *p* < 0.001 |
| BS1 |  | *p* < 0.001 | *p* < 0.001 | *p* < 0.001 |
| BS2 |  | *p* < 0.001 | *p* < 0.001 | *p* < 0.001 |
| BS3 |  | *p* < 0.001 | *p* < 0.001 | 0.720 |
| BS4 |  | *p* < 0.001 | *p* < 0.001 | 0.180 |
| BS5 |  | *p* < 0.001 | *p* < 0.001 | *p* < 0.001 |
| BS6 |  | *p* < 0.001 | *p* < 0.001 | 0.001 |
| BS7 |  | *p* < 0.001 | *p* < 0.001 | *p* < 0.001 |
| BS8 |  | *p* < 0.001 | *p* < 0.001 | *p* < 0.001 |
| BS9 |  | *p* < 0.001 | *p* < 0.001 | *p* < 0.001 |
| BS10 |  | *p* < 0.001 | *p* < 0.001 | *p* < 0.001 |
| BS11 |  | *p* < 0.001 | *p* < 0.001 | *p* < 0.001 |
| BS12 |  | *p* < 0.001 | *p* < 0.001 | 1.000 |
| BS13 |  | *p* < 0.001 | *p* < 0.001 | 0.020 |
| BS14 |  | *p* < 0.001 | *p* < 0.001 | *p* < 0.001 |
| N1 |  | *p* < 0.001 | *p* < 0.001 | *p* < 0.001 |
| N2 |  | *p* < 0.001 | *p* < 0.001 | *p* < 0.001 |
| N3 |  | *p* < 0.001 | *p* < 0.001 | *p* < 0.001 |
| N4 |  | *p* < 0.001 | *p* < 0.001 | 1.000 |
| N5 |  | *p* < 0.001 | *p* < 0.001 | *p* < 0.001 |
| N6 |  | *p* < 0.001 | *p* < 0.001 | 0.001 |
| D1 |  | *p* < 0.001 | 0.001 | *p* < 0.001 |
| D2 |  | *p* < 0.001 | *p* < 0.001 | 0.310 |
| D3 |  | *p* < 0.001 | *p* < 0.001 | *p* < 0.001 |
| D4 |  | *p* < 0.001 | 0.220 | *p* < 0.001 |
| G1 |  | *p* < 0.001 | *p* < 0.001 | 0.011 |
| G2 |  | *p* < 0.001 | *p* < 0.001 | 0.003 |
| G3 |  | *p* < 0.001 | *p* < 0.001 | 0.011 |
| G4 |  | *p* < 0.001 | *p* < 0.001 | *p* < 0.001 |
| Verbal Memory |  | *p* < 0.001 | 0.022 | 0.386 |
| Processing Speed |  | *p* < 0.001 | 0.030 | 0.031 |
| (Verbal) executive functions |  | *p* < 0.001 | 0.569 | 0.008 |
| Affective disorders |  | 0.003 | *p* < 0.001 | 0.914 |
| Anxiety disorders |  | 0.116 | 0.006 | 0.856 |
| Post-traumatic stress disorders |  | 0.161 | 0.013 | 0.871 |
| Eating disorders |  | 0.652 | *p* < 0.001 | 1.000 |
| Substance addiction |  | 0.412 | 0.248 | 0.955 |
| Substance abuse |  | 0.124 | 0.172 | 0.398 |
| Transition to psychosis |  | *p* < 0.001 | *p* < 0.001 | *p* < 0.001 |

*Note.* ^a^ Post-hoc test could not be computed due to count of cells being smaller than 5

**Supplemental Table 6. Description of Sample across Centers**

|  | FETZ Bern^a^  (n = 257) | BEARS-Kid^b^  (n = 461) | FETZ Cologne^c^  (n = 157) | Statistics, effect size | Significant post-hoc tests, Bonferroni corrected |
| --- | --- | --- | --- | --- | --- |
| Age, M (SD) | 18.9 (4.63) | 14.68 (2.41) | 24.71 (5.57) | *F*(1, 873)=57.51, *p*<0.001, η^2^=0.06 | a vs. b; a vs. c; b vs. c |
| Sex, n (% Female) [SR] | 117 (45.5) | 266 (57.7)  [4.56] | 58 (36.9)  [-3.72] | χ^2^_(2)_=23.65, *p*<0.001, V=0.16 |  |
| Nationality, n (%) [SR] |  | | | | |
| Swiss | 224 (87.2)  [12.38] | 256 (55.5) | 0 (0.0)  [-15.25] | χ^2^_(8)_=358.93, *p*<0.001, V=0.45 |  |
| German | 1 (0.4)  [-13.80] | 165 (35.8) | 139 (88.5)  [15.58] |  |  |
| Other | 32 (12.4) | 40 (8.7)  [-2.00] | 18 (11.5) |  |  |
| Any Axis 1 Disorder, n (%) [SR] | 171 (69.0)  [-5.61] | 398 (86.5)  [5.61] | - | χ^2^_(1)_=30.42, *p*<0.001, V=0.21 |  |

*Note.* MINI/MINI-Kid data not available for the FETZ Cologne; Cramer’s V interpretation: 0.1=weak effect; 0.3=moderate effect; 0.5=strong effect; Partial η2: 0.01=weak effect; 0.06=moderate effect; 0.14=strong effect; SR=standardized residual; only presented for cells with SR of ≥|1.96|, which equals significant deviation from the expected cell frequency, with positive values indicating higher-than-expected cell frequency and negative values indicating lower-than-expected cell frequency.

**Supplemental Table 7. Description of Study Centers**

|  | **FETZ Bern** | **BEARS-Kid** | **FETZ Cologne** |
| --- | --- | --- | --- |
| Study center | Clinical center that targets help-seeking individuals with putative psychotic symptoms or CHR symptoms | Help-seeking patients of one of the early detection centres in Bern, Zurich or Cologne; a non-psychotic clinical sample with a DSM-IV diagnosis for which an increased prevalence of subsequent schizophrenia has been reported | Clinical center for early detection of psychosis |
| Inclusion/  Exclusion criteria | - Given informed consent - Age between 8-40 - No past clinical diagnosis of any psychotic disorder according to the DSM and ICD - No diagnosis of delirium, dementia, amnestic or other neurological disorders - No general medical conditions affecting the central nervous system. - Insufficient language (German, French or English) skills | - Given informed consent from participants and parent - Age between 8-17 years - No lifetime diagnosis of psychosis - Insufficient language (German, French or English) skills - For clinical sample the following diagnoses: ADHD, Social Phobia, Specific Phobia, OCD, Anroexia nervosa, Bulimia nervosa, and Asperger’s Disorder | - Given informed consent - Age between 16-40 - No past clinical diagnosis of any psychotic disorder according to the DSM or ICD - No diagnosis of delirium, dementia, amnestic or other neurological disorders, no psychiatric disorders due to a somatic factor or related to psychotropic substances - No general medical conditions affecting the central nervous system |
| Recruitment period | Ongoing since 2009 | 33 months (from September 2011 to May 2014) | 1998 until end of 2003 |
| Transition rate, n (%) | 0 (0.00) | 13 (2.82) | 59 (37.58) |
| Time to transition in months, M (SD), Mdn, Min-Max |  | 13.18 (12.42), 7.36, 2.30-38.11 | 15.05 (12.65), 9.63, 0.36-50.30 |
| Website | <https://www.upd.ch/de/angebot/kinder-und-jugendpsychiatrie/spezialangebote/fetz.php> | <https://www.kjpd.uzh.ch/de/klinische-forschung/psychose/projects/bears.html> | <https://psychiatrie-psychotherapie.uk-koeln.de/klinik/frueherkennungs-und-therapiezentrum-fetz/> |

*Note.* Time to transition not available for two patients from the BEARS-Kid
